# Depression, anxiety and self-esteem in adolescent girls with polycystic ovary syndrome: a systematic review and meta-analysis

**DOI:** 10.3389/fendo.2024.1399580

**Published:** 2024-09-30

**Authors:** Yuxin Li, Jiayu Zhang, Xuanling Zheng, Wenjing Lu, Jinru Guo, Fuhong Chen, Changqin Liu

**Affiliations:** ^1^ Department of Endocrinology and Diabetes, The First Affiliated Hospital of Xiamen University, School of Medicine, Xiamen University, Xiamen, China; ^2^ Department of Nursing, The First Affiliated Hospital of Xiamen University, School of Medicine, Xiamen University, Xiamen, China; ^3^ Xiamen Key Laboratory of Clinical Efficacy and Evidence Studies of Traditional Chinese Medicine, The First Affiliated Hospital of Xiamen University, School of Medicine, Xiamen University, Xiamen, China

**Keywords:** polycystic ovary syndrome, adolescent, depression, anxiety, self-esteem

## Abstract

**Objective:**

Studies have shown the adverse psychological impact of polycystic ovary syndrome (PCOS), but the state of mental health in adolescents with PCOS remains unclear. Thus, we performed a systematic review and meta-analysis to investigate the prevalence and severity of depression and anxiety, as well as potential effects on self-esteem and quality of life (QoL) in this specific population.

**Methods:**

We systematically searched four electronic databases: PubMed, Embase, Web of Science, and the Cochrane Reviews database for articles published until 25/8/2024. We considered observational studies in which the subjects were adolescent girls with PCOS who had reported symptoms including anxiety, depression, self-esteem, and QoL. The Review Manager version 5.4 was used to analyze the available data extracted. We used the Newcastle-Ottawa Quality Assessment Scale (NOS) to evaluate the quality of selected studies. A funnel plot was utilized to assess the risk of literature bias, and a forest plot was used to represent the combined outcomes. This systematic review was previously registered in PROSPERO with the registration number CRD42022382036.

**Results:**

We included 11 studies in the systematic review and conducted meta-analyses on 10 of them. Adolescents with PCOS reported a higher risk of depression (OR = 2.21, 95% CI: 1.23 to 4.00, p = 0.008) and a higher level of depression scores (SMD = 0.43, 95% CI: 0.16 to 0.71, p = 0.002) than controls. There were no significant differences in anxiety (OR = 1.90, 95% CI: 0.52 to 6.96, p = 0.33; SMD = 0.19, 95% CI: -0.21 to 0.59, p = 0.36), self-esteem (SMD = -0.17, 95% CI: -0.85 to 0.52, p = 0.64), and QoL (SMD = -0.15, 95% CI: -0.42 to 0.11, p = 0.26) between the two groups.

**Conclusions:**

Our research indicated that adolescents with PCOS experienced more severe depressive symptoms than those without PCOS. This highlights the importance of evaluation and early treatment of mental health in PCOS. More clinicians should pay attention to the mental health of adolescent girls with PCOS through this study.

**Systematic Review Registration:**

https://www.crd.york.ac.uk/PROSPERO/, identifier CRD42022382036.

## Introduction

Polycystic ovary syndrome (PCOS), a prevalent endocrine-metabolic disease in women of reproductive age, usually appears in adolescence (1). Its prevalence ranges from 5% to 18% in women and 3.4% to 11% in adolescent girls, depending on different diagnostic criteria (2, 3). The primary clinical characteristics encompass irregular menstrual periods, presentation of androgen excess (such as hirsutism, acne, and laboratory hyperandrogenism), and polycystic ovary morphology (PCOM) on ultrasound. According to the commonly used Rotterdam criteria, meeting at least 2 of the above criteria and excluding other diseases can be diagnosed as PCOS (4). However, the diagnosis of PCOS in adolescent girls remains challenging due to the overlap of these features with the presentation in adolescence. Based on the recent 2023 Evidence-Based Guidelines for PCOS, menstrual irregularity and clinical/biochemical hyperandrogenemia are necessary to diagnose PCOS in adolescents, and PCOM is not suggested as a diagnostic criterion for PCOS in adolescence (5).

Women with PCOS are at significant risk of a broad spectrum of complications, including infertility, obesity, insulin resistance, metabolic syndrome, diabetes, and cardiovascular disease (6). In addition to the metabolic and reproductive aspects, PCOS significantly impacts the psychological health of women affected (6). It should be noted that adolescents are vulnerable to mental health problems, given the dramatic changes in hormones, body, brain, social environment, and cognition in this special period (7). For adolescent girls with PCOS, the illness’s underlying pathophysiology and associated physical features may lead to apparent concerns about body image, reduction of self-esteem, depression, and anxiety (8–10). Depression is distinguished by a consistently low mood and a lack of interest or pleasure, with primary symptoms including appetite disorders, sleep disturbances, and concentration deficit, among others (11). In recent years, many cross-sectional studies have investigated the fact that the prevalence of depression has significantly increased in girls with PCOS. Anxiety is an emotional experience of inner nervousness and impending disaster, often manifested as excessive concern, inability to sit still, and autonomic nervous disorders, such as chest tightness, palpitations, sweating, and so on (12). Self-esteem is an individual’s subjective evaluation of self-worth, and mental disorders are related to the decline of self-esteem (13, 14). There is growing evidence that women with PCOS are prone to psychological problems. For these patients, the quality of life (QoL) can be substantially impacted in various aspects of fulfilling life and subjective well-being (15). Besides, adolescent depression and anxiety are linked to elevated odds of adult depression and anxiety disorders, thereby adding to the long-term burden of disease (16, 17). Considering elements like the greater possibility of binge eating, higher sedentary behaviors, and the use of psychiatric medications, these psychological issues are likely to contribute to weight gain and a higher risk of cardiovascular disease (18, 19). In this sense, psychological disorders in adolescence can have multiple, long-term detrimental effects on the well-being of patients.

At present, several researchers have conducted investigations on depression and anxiety in adolescent girls with PCOS. Emeksiz HC. et al. reported that female adolescents with PCOS exhibited higher levels of depression and anxiety compared to healthy controls (20). In a cross-sectional study conducted by Çoban ÖG and colleagues, the rate of psychiatric disorders, including major depressive disorder and anxiety disorder, in the PCOS group was considerably higher than that in the control subjects (21). However, Zachurzok A. et al. had a contradictory result, which showed that no association was discovered between self-esteem, anxiety, depression, and PCOS in adolescent girls (22). Collectively, the findings of studies on this topic are still inconclusive. Furthermore, to our knowledge, no meta-analysis has been performed on anxiety and depression in adolescent girls with PCOS.

Given the numerous adverse impacts of psychological problems and the contrasting conclusions regarding the mental health of adolescent girls with PCOS, it is critical to summarize available evidence in this area. Therefore, we undertook a systematic review and meta-analysis of observational studies that explored depression and anxiety of adolescent females with PCOS. This study aimed to investigate the prevalence and extent of depression and anxiety, as well as the situation of self-esteem and QoL in adolescent girls with PCOS. We anticipate that this research will be helpful for clinicians in timely identifying high-risk populations and promoting the physical and mental well-being of women with PCOS.

## Methods

### Search strategy

This meta-analysis was conducted by the PRISMA guidelines (23) (**Supplementary Tables S1**, **S2**). We systematically searched for published studies up to 25/8/2024 in the four electronic databases: PubMed, Embase, Web of Science, and the Cochrane Reviews. The search strategy used was a combination of subject headings and free words for PCOS, adolescents, and depression/anxiety (including depression, anxiety, mental disorders, mood disorders, psychological distress, mental health, and emotion). Detailed search strategies were provided in the **Supplementary Materials** (**Supplementary Data Sheet 1**). The PICOS framework for systematic review: for patients with PCOS (Participants), depression, anxiety, self-esteem, and QoL (Outcomes) for the observational group than girls without PCOS (Comparison) in observational studies (Type of studies). We also manually retrieved the references of relevant review articles for potentially eligible studies. Additionally, we looked through the relevant gray literature and dissertations and, when necessary, contacted the study authors to request additional information. The systematic review was previously registered in PROSPERO with the registration number CRD42022382036.

### Study selection

Studies that met the following criteria were included: (1) the study was a case-control, longitudinal cohort, or cross-sectional design with one group of subjects with PCOS and another group without PCOS; (2) the study population was between 10 and 19 years old [according to WHO (24)]; (3) the study reported the rate or level of depression/anxiety or depressive/anxiety symptoms using validated assessment tools, whether rating scales, diagnostic interviews or clinical diagnosis; and (4) the article was published in English. Studies published as reviews and abstracts were excluded owing to the lack of comprehensive information, such as detailed study protocols.

### Data extraction

Two authors independently reviewed and checked the eligibility of studies and extracted relevant data, with any discrepancies resolved by a third author. The following general information was obtained from each included study: publication information (first author, publication year, country of publication, and quality assessment); study characteristics (study design, recruitment of participants, sample size, diagnostic criteria for PCOS, reported outcomes, and used screening tools); subjects’ information (range of age, mean age, mean body mass index, and matched variables between PCOS group and control group).

### Study outcomes

Regarding outcomes, we took depression and anxiety as the primary indices and self-esteem and QoL as the secondary index. We extracted the number of related events and the mean and standard deviation of scores as accurately as possible. Given that trait anxiety refers to consistent emotional experience and state anxiety refers to transient emotional response, we gave priority to the score of the trait anxiety subscale in the State-Trait Anxiety Inventory (25). Besides, because there was no significant cutoff value in the study of Almis H. and colleagues, we defined a trait anxiety score of less than 40 as a normal level according to the scoring rule (26).

### Quality assessment

We used the Newcastle-Ottawa Quality Assessment Scale (NOS) to evaluate the quality of selected studies, with a maximum score of 9 (27). According to the scale, 0-3 scores indicated low quality, 4-6 scores indicated moderate quality, and 7-9 scores indicated high quality. Two independent reviewers conducted the quality assessment, and a third author addressed disagreements.

### Statistical analysis

All analyses were performed using Review Manager version 5.4. The prevalence of depression and anxiety was calculated using odds ratios (OR) and 95% confidence intervals (CI). The effect sizes of the mean difference in depression, anxiety, self-esteem, and QoL between PCOS subjects and controls were calculated using the standardized mean difference (SMD) and 95% CI. The I^2^ statistic was used to assess heterogeneity among studies. When heterogeneity was high (I^2^>50%), we used the random effects model to estimate pooled effect size; otherwise, we used the fixed effects model (28). Subgroup analyses were utilized to explore the reason for heterogeneity. A funnel plot was used to evaluate the risk of literature bias. P<0.05 was considered statistically significant in all analyses.

## Results

### Literature selection

**Figure 1:**
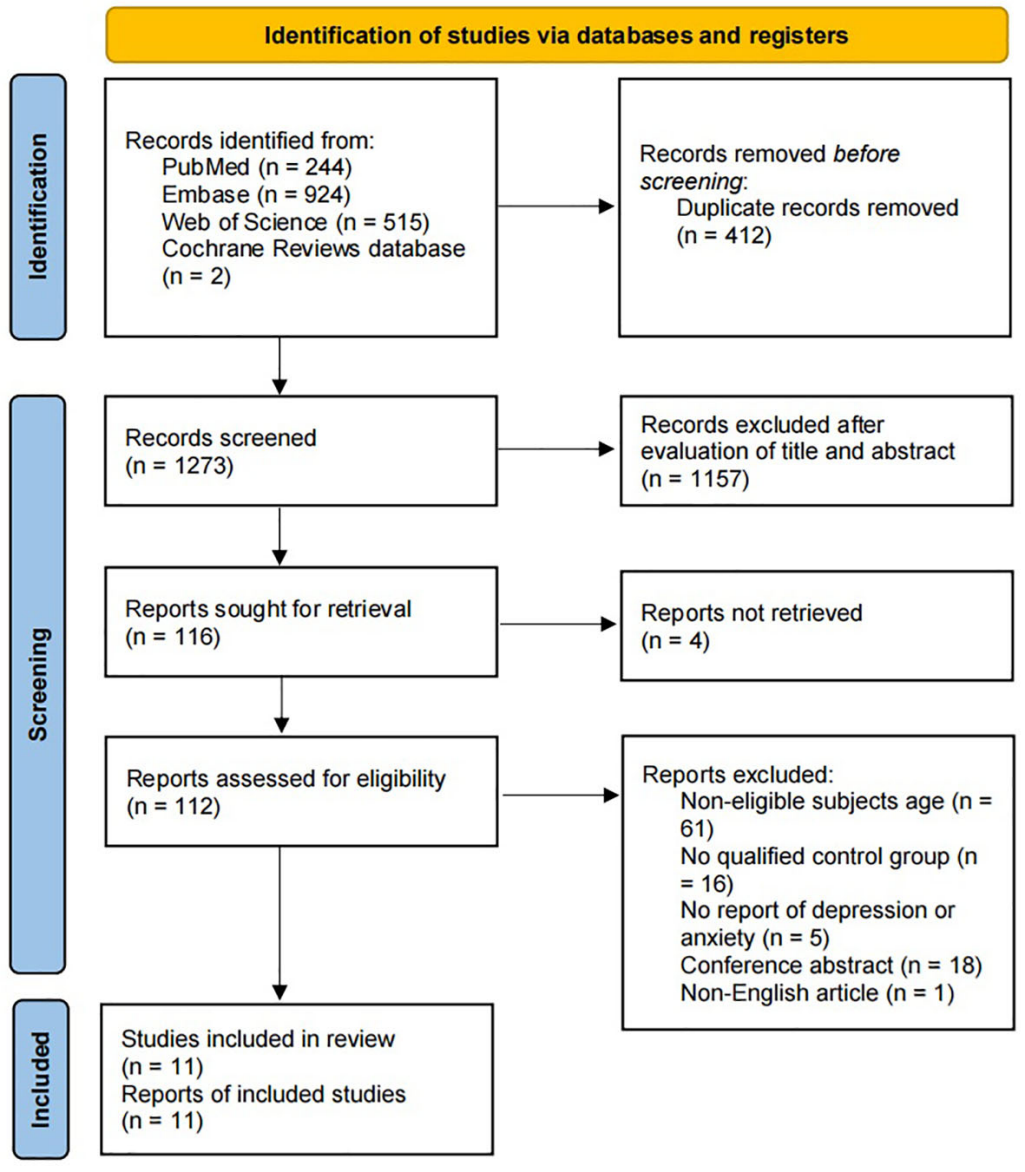
Flow diagram of study selection.

**Figure 1** shows the flow diagram for study selection. Our systematic literature search identified 1,685 potentially relevant records. After removing duplicate articles and screening by title and abstract, 116 articles remained for further full-text review. 105 studies were excluded for the reasons illustrated in **Figure 1**, and 11 were eligible according to our predetermined inclusion criteria. However, the results in one of the studies were not described in terms of mean and standard deviation and were not included in our meta-analysis.

### Study characteristics

**Table 1** summarizes the characteristics of 11 eligible studies, which comprised 545 girls with PCOS and 2,042 controls (20–22, 26, 29–35). These studies were published from 2013 to 2022, including nine cross-sectional and two cohort studies. Although six studies were performed in Turkey, there were also five studies from Lebanon, New Zealand, Italy, the United States of America (USA), and Poland, respectively. Nearly all the studies matched PCOS and control groups by age, and four matched body mass indexes (BMI). The quality assessment scores for the included studies ranged from 2 to 7, with a mean score of 4.91.

**Table 1:** Characteristics of included studies.

First author	Year	Study design	Country	Recruitment of participants (case/control)	Range of age	Sample size (case/control)	Mean age (case/control)	Mean BMI (case/control)	PCOS definition	Reported outcomes	Screening tools	Variables matched	Quality assessment
Ghazeeri, G.	2013 (29)	Cross-sectional	Lebanon	Both: hospital	14-18	20/17	16.7(1.1)/ 16.4 (1.3)	26.3(4.2)/ 21.1(2.55)	Rotterdam criteria	depression, anxiety	BDI, SCARED	age, father’s education level	5
Milsom, S. R.	2013 (30)	Cohort	New Zealand	P: hospital C: school	14-19	66/1349	Both: NA	NA/ 21.8(14.9-62.5)	Rotterdam criteria	depression	RADS-2	age, ethnicity, deprivation index	6
Guidi, J.	2015 (31)	Cross-sectional	Italy	Both: school	16-19	17/240	17.6(0.9)/ 17.4 (0.9)	23.4(5.0)/ 21.7(3.1)	The combination of menstrual irregularity with clinical hyperandrogenism and/or hyperandrogenemia, after the exclusion of specific etiologies	depression, anxiety, quality of life	SQ, PSI	age, BMI	7
Emeksiz, H. C.	2018 (20)	Cross-sectional	Turkey	P: hospital C: general population	16-19	80/50	17.23(1.15)/ 17.00(0.99)	24.7(21.0-29.6)/ 24.3(20.1-27.3)	Rotterdam criteria	depression, anxiety	SCARED, CDI	age, BMI	7
Çoban Ö, G.	2019 (21)	Cross-sectional	Turkey	P: NA C: school	13-19	28/31	Both: NA	22.68(5.21)/ 20.06(1.83)	NA	depression, anxiety, quality of life, self-esteem	SADS-PL, RSES, PedsQL	age	4
Benson, J.	2020 (32)	Cohort	USA	Both: hospital	11-17	14/44 (PCOS+T2D/T2D)	14.5(13.9-16.3)/14.51 (12.5-16.5)	34.74(31.4-39.7)/ 34.8(29.6-42.6)	Oligomenorrhea > 2 years post menarche and clinical/biochemical hyperandrogenism, with no other cause of oligomenorrhea or elevated androgens noted	depression	CES-D	NA	2
Sari, S. A.	2020 (33)	Cross-sectional	Turkey	Both: hospital	12-18	50/37	16.01(1.19)/ 16.00(1.49)	26.18(5.55)/ 22.07(4.05)	Persistent oligomenorrhea beyond 2 years after menarche (menstrual cycles > 45 days), clinical and/or biochemical hyperandrogenemia, and exclusion of secondary causes of hyperandrogenism	depression, anxiety, self-esteem	KSADS-PL, CDI, RSES	age	4
Almis, H.	2021 (26)	Cross-sectional, Case-control	Turkey	Both: hospital	13-18	153/161	15.57(1.11)/ 15.71(1.05)	21.84(3.47)/ 20.53(2.75)	Rotterdam criteria	depression, anxiety	CDI, STAI-C	age	4
Besenek, M.	2021 (34)	Cross-sectional, Case-control	Turkey	Both: hospital	11-18	39/37	17.0(IQR:2)/ 17.5(IQR:2)	24.3(5.71)/ 19.6(3.03)	Irregular menses (oligo-amenorrhea: menstrual cycles > 45 days) and hyperandrogenism > 2 years after menarche	depression, anxiety	BDI, STAI	age	5
Zachurzok, A.	2021 (22)	Cross-sectional	Poland	Both: hospital	13-18	27/27	16.7(1.2)/ 16.1(1.1)	z-score: 1.1(0.9)/ 1.0(1.0)	Menstrual disturbances (oligomenorrhea, secondary amenorrhea) and clinical or biochemical hyperandrogenism	depression, anxiety, self-esteem	HADS, RSES	age, BMI	5
Donbaloğlu, Z.	2022 (35)	Cross-sectional, Case-control	Turkey	Both: hospital	13-18	51/49	15.72(1.32)/ 15.53(1.77)	24.3(5.1)/ 20.6(2.48)	Menstrual irregularity (oligomenorrhea: menstrual cycle > 45 d) and hyperandrogenism at least 2 yr since menarche	depression, anxiety, quality of life	BDI, STAI, PedsQL	age	5

BMI, body mass index; IQR, interquartile range; BDI, The Beck Depression Inventory; SCARED, The Screen for Child Anxiety Related Emotional Disorders; RADS-2, The Reynolds Adolescent Depression Scale; SQ, The Symptom Questionnaire; PSI, The Psychosocial Index; CDI, The Child Depression Inventory; SADS-PL, Schedule for Affective Disorders and Schizophrenia for School Age Children Present and Lifetime; RSES, The Rosenberg Self-Esteem Scale; PedsQL, The Pediatric Quality of Life Inventory; CES-D, Center for Epidemiologic Studies-Depression; KSADS-PL, Kiddie Schedule for Affective Disorders and Schizophrenia for School-Aged Children-Present and Lifetime Version; STAI-C, The State-Trait Anxiety Inventory for Children; STAI, The State-Trait Anxiety Inventory; HADS, Hospital Anxiety and Depression Scale

Regarding outcomes, all studies reported depression/depressive symptoms; nine studies reported anxiety/anxiety symptoms; three studies reported QoL and three studies reported self-esteem by applying multiple valid evaluation tools. The depressive symptoms were measured based on rating scales including the Beck Depression Inventory (29, 34, 35), the Child Depression Inventory (20, 26, 33), Hospital Anxiety and Depression Scale (22), the Symptom Questionnaire (31), the Center for Epidemiologic Studies-Depression (32), and the Reynolds Adolescent Depression Scale (30). The anxiety symptoms were assessed based on scales including the State-Trait Anxiety Inventory (26, 34, 35), Hospital Anxiety and Depression Scale (22), the Symptom Questionnaire (31), and the Screen for Child Anxiety Related Emotional Disorders (20, 29). In two studies, the psychiatric disorders were evaluated by the Schedule for Affective Disorders and Schizophrenia for School Age Children Present and Lifetime (21, 33).

### Meta-analysis

#### Depression

**Figure 2:**
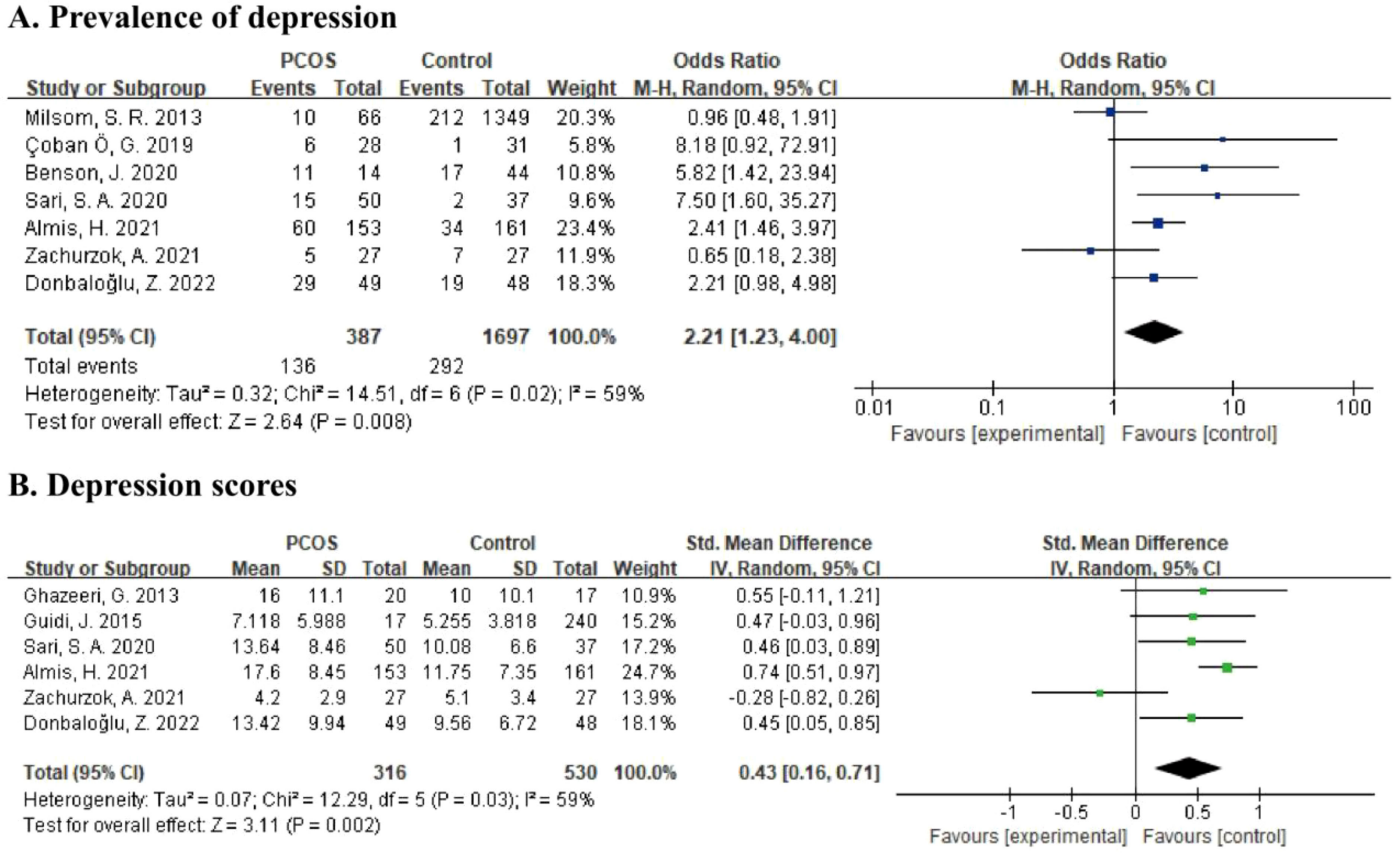
**(A)** Forest plot comparing the prevalence of depression between PCOS group and control group. **(B)** Forest plot of depression scores in PCOS group and control group.

For the prevalence of depression, data from seven studies comprising 2,084 participants was calculated in the meta-analysis (21, 22, 26, 30, 32, 33, 35) (**Figure 2A**). The results showed a pooled OR of 2.21 (95% CI: 1.23 to 4.00, p = 0.008) based on the random effects model, which indicated that adolescent girls with PCOS were 2.21 times more likely to have depression than subjects without PCOS. Besides, the heterogeneity of this comparison was moderate at 59% (p = 0.02).

For the severity of depressive symptoms, six studies reporting depression scores involving 846 subjects were included in meta-analysis (22, 26, 29, 31, 33, 35) (**Figure 2B**). By using the random effects model, there was a pooled SMD of 0.43 (95% CI: 0.16 to 0.71, p = 0.002) with moderate heterogeneity (I^2^ = 59%, p = 0.03), which showed that a higher level of depression was present in PCOS adolescents than in controls.

#### Anxiety

**Figure 3:**
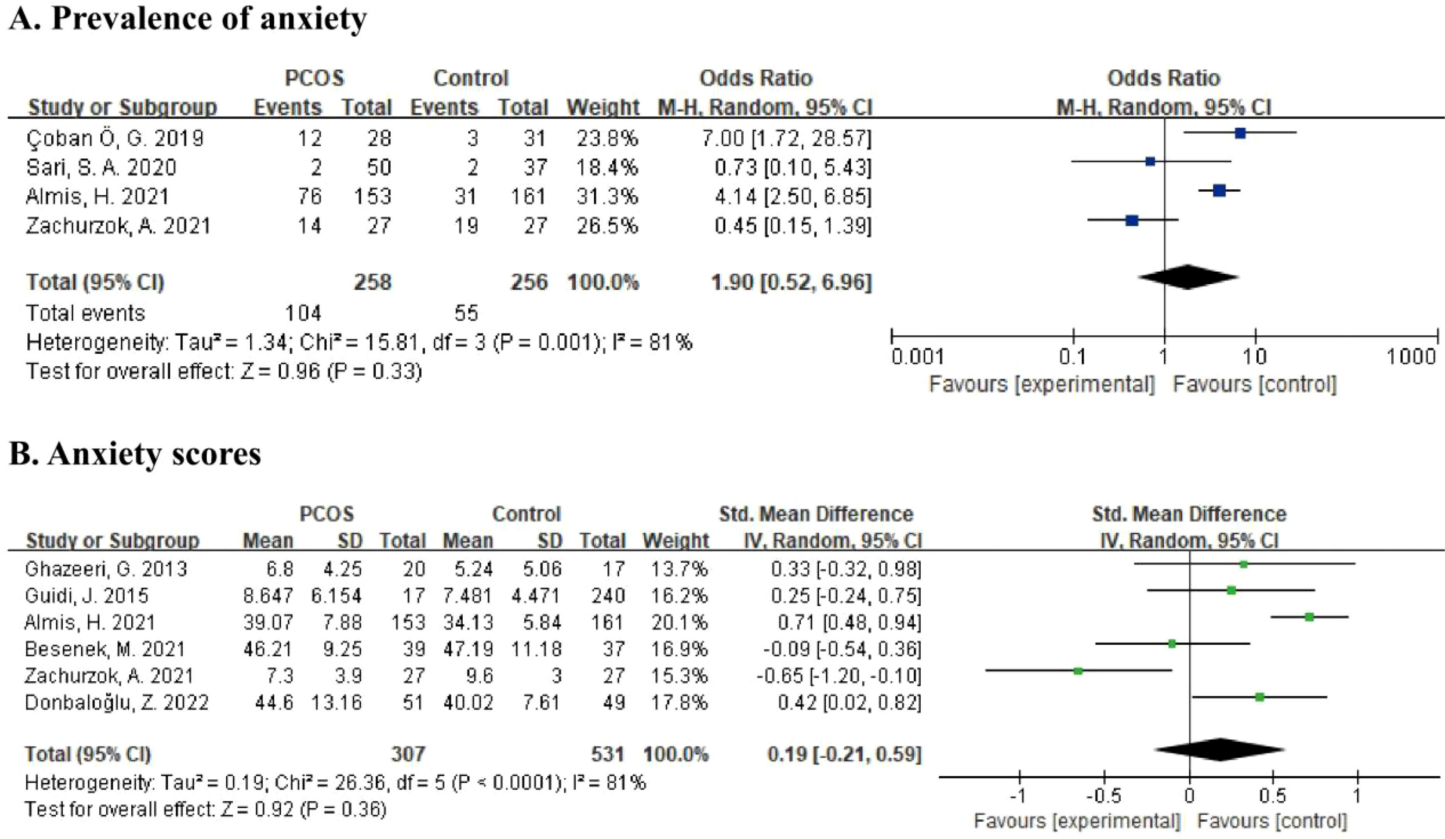
**(A)** Forest plot comparing the prevalence of anxiety between PCOS group and control group. **(B)** Forest plot of anxiety scores in PCOS group and control group.

Data from four studies, including 514 participants, was calculated in the meta-analysis21,22,26,33 (**Figure 3A**) for the prevalence of anxiety. According to the random effects model, the pooled OR was 1.90 (95% CI: 0.52 to 6.96, p = 0.33), and the results were highly heterogeneous (I^2^ = 81%, p = 0.001). For the severity of anxiety symptoms, six studies providing anxiety scores with a total of 838 subjects were included in the meta-analysis (22, 26, 29, 31, 34, 35) (**Figure 3B**). Using the random effects model, the pooled SMD was estimated as 0.19 (95% CI: -0.21 to 0.59, p = 0.36). Heterogeneity among studies was relatively high (I^2^ = 81%, p < 0.0001).

#### Self-esteem

**Figure 4:**
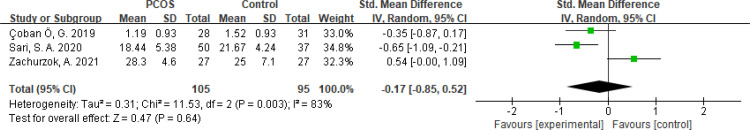
Forest plot of self-esteem scores in PCOS group and control group.

Of all qualified studies, three articles examined the self-esteem status in the PCOS and control groups (21, 22, 33). **Figure 4** shows no significant difference in self-esteem scores between the two groups (SMD = -0.17, 95% CI: -0.85 to 0.52, p = 0.64; I^2^ = 83%, p = 0.003).

#### QoL

**Figure 5:**
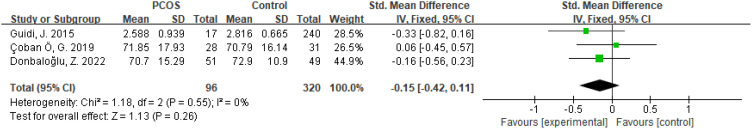
Forest plot of quality of life scores in PCOS group and control group.

Of all eligible studies, three investigated the QoL situation between the PCOS group and controls (21, 31, 35). As illustrated in **Figure 5**, the two groups had no significant difference in QoL scores (SMD = -0.15, 95% CI: -0.42 to 0.11, p = 0.26; I^2^ = 0%, p = 0.55).

### Subgroup analyses

In subgroup analyses by continent of origin (**Supplementary Figure S1**), results remained significant for depression in the Asian group, with higher prevalence and scores in PCOS versus controls. The values of I^2^ in the Asian group were substantially decreased, which implied that the region might be a potential source of heterogeneity. However, there was still high heterogeneity in the results of anxiety, both in the Asian and non-Asian groups. In subgroup analyses by screening tool (**Supplementary Figure S2**), results were still significant in the BDI (the Beck Depression Inventory) group and CDI (the Child Depression Inventory) group with minor heterogeneity, which implied that differences in screening tools might partly explain the heterogeneity. However, there was still high heterogeneity in the results of anxiety scores in both subgroups.

## Discussion

This is the first systematic review and meta-analysis to summarize available evidence on the situation of depression and anxiety in adolescent girls (aged 10 - 19 years old) with PCOS. In this meta-analysis, we discovered that adolescent females with PCOS had a higher risk of depression and a higher level of depression scores than controls. However, there were no significant differences in the prevalence and extent of anxiety between the two groups. In addition, self-esteem and QoL, the secondary indices in our study, were also not substantially different between the two groups.

In the current study, PCOS adolescents were 2.21 times more likely to have depression than the control group. In line with our results, the statistically significant association between PCOS and depression was also observed in some previous meta-analyses mainly targeting adults, with elevated odds of depression over two to four times in PCOS participants (36–39). Besides, we found that PCOS adolescents experienced more serious depressive symptoms than controls, which was also in accordance with previous studies (39–42). Even though our effect sizes are lower than those in all of the research mentioned above, which primarily focused on adult PCOS, it is still important to note the significant link between PCOS in adolescents and depression. Based on global estimate, approximately 280 million people are affected by depression, with its prevalence rises significantly during adolescence (43, 44). Extensive evidence has illustrated that early-life depression is linked to several adverse health outcomes, including not only suicidal tendencies, recurrence and co-occurrence of mental disorders, and increased risk of cardiovascular disease but also long-term impairments in education, employment, and social participation (16, 18, 45). As a result, our study highlighted the need to early identify and manage depression in adolescents with PCOS to foster favorable health outcomes.

**Figure 6:**
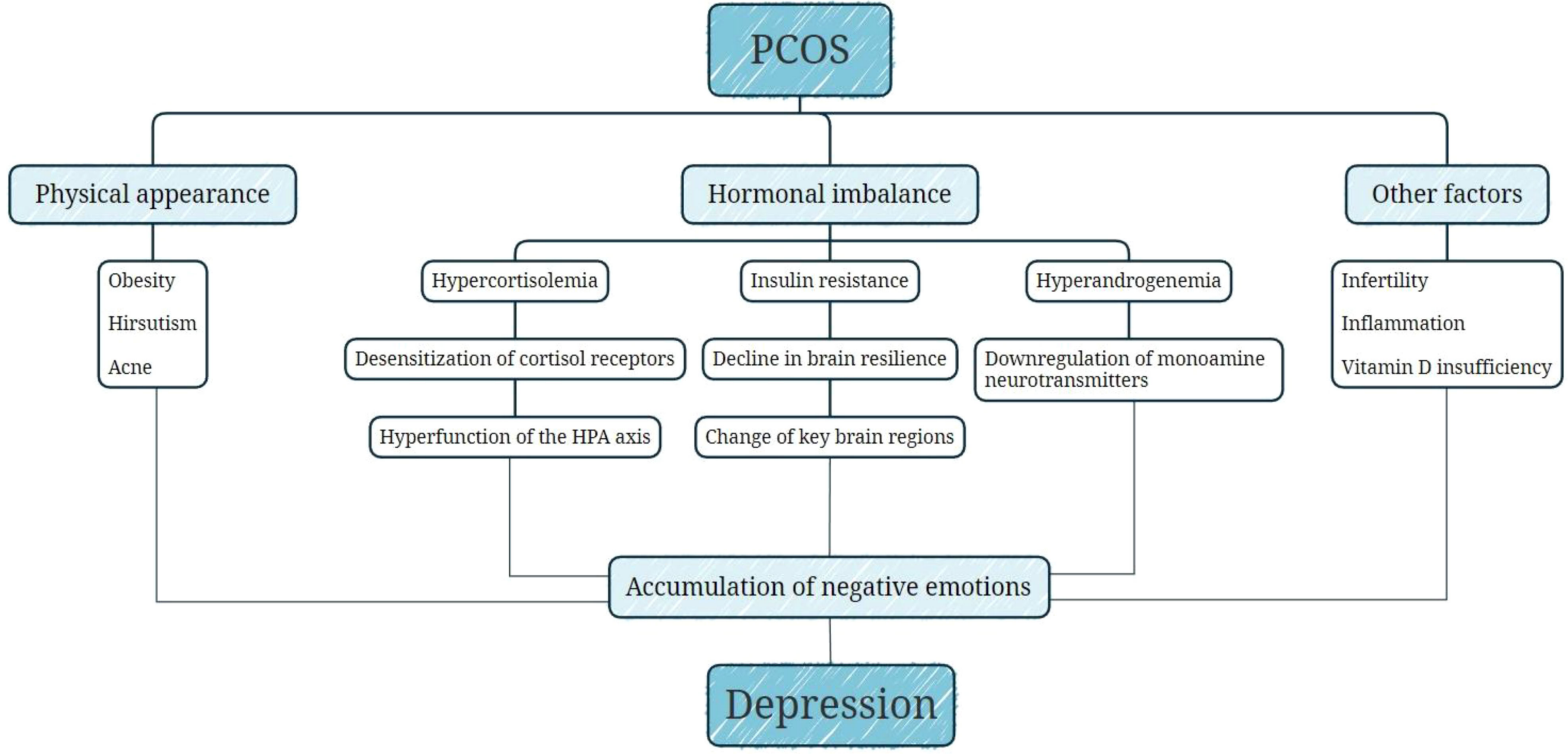
Potential mechanisms between PCOS and depression.

The mechanisms underlying the connection between PCOS and vulnerability to depression are not fully clarified, and several potential factors have been explored (**Figure 6**). Women with PCOS may be distressed about the changes in physical appearance caused by obesity and hirsutism, which become a possible source of PCOS women’s depressed emotion (9, 36). Additionally, their subjective perceptions of symptoms play a more critical role than objective assessment (46). Besides, some PCOS patients’ concerns regarding infertility place a specific psychological strain on them (47). From the point of view of pathophysiology, the potential contribution of hormonal imbalance has also been investigated. Hypercortisolemia caused by chronic stress and hyperactivity of the hypothalamic-pituitary-adrenal (HPA) axis plays a crucial role in the development of depression (48). It has been shown that women with PCOS experience a more significant increase in cortisol levels after exposure to stress compared to controls (49). Persistently high cortisol levels will lead to desensitization of cortisol receptors and failure of negative feedback regulation, resulting in persistent hyperfunction of the HPA axis and a vicious cycle (50). Insulin resistance may be associated with depression in PCOS, serving as a physiologic mediator (51). In a randomized controlled trial, the increase of the homeostatic model of insulin resistance (HOMA-IR) elevated the odds of depression by 2.32 (51). One possible explanation is that insulin resistance makes the brain less adaptable, which can lead to changes in the structure or function of key brain regions to influence emotion regulation (52). In addition, in an experiment, dehydroepiandrosterone-induced PCOS mice exhibited depression-like behavior, possibly by down-regulating monoamines and/or their metabolites in brain (53). Hyperandrogenemia may be related to depression in PCOS, which needs to be further explored in human studies. Moreover, several relevant mechanisms have been discussed: inflammation (54), vitamin D insufficiency (55), and change of neurotransmitter (54, 56), which may also be involved in the development of depression in PCOS. Several studies have confirmed the link between potential factors mentioned above [such as BMI (20), hirsutism (26), acne (26), and free testosterone levels (35)] and depression in the adolescent PCOS population.

In our study, no association was observed between PCOS and anxiety in adolescent populations. Although none of the previous meta-analyses on this topic focused entirely on adolescents, the results of subgroup analyses based on age in several studies provided relevant evidence. A meta-analysis conducted by Veltman-Verhulst SM and colleagues suggested that there was no significant difference in anxiety scores within the subgroup of PCOS women who were 24 years old or younger (40). Brutocao C. et al. found that mixed adult/pediatric populations with PCOS had a 1.70-fold risk of anxiety disorder compared to controls, which was lower than in the adult group (OR= 5.22) (39). Taking our results together with previous evidence, we speculate that PCOS has not yet had a notable effect on the situation of anxiety in adolescent girls. This may be because adolescents are still in the early stage of PCOS, and the duration of symptoms is relatively short. What’s more, some symptoms of PCOS, such as irregular menstruation, are likely to be considered normal in adolescence and not enough to attract the attention of adolescents (57). Nonetheless, it should be noted that a lack of included studies constrains the existing evidence. Therefore, our conjecture needs further verification.

In the present study, no substantial differences remained in the domains of self-esteem and QoL between the adolescent PCOS group and the control group. Self-esteem comes from a positive evaluation of self-worth; for teenagers, appearance is an important factor affecting self-esteem (58, 59). Although PCOS may be accompanied by some physical changes, no significant effect of the illness on adolescent girls’ self-esteem was observed in our study. In terms of QoL, only one study has conducted a review targeting adolescents with PCOS, and most of the studies in this review showed impaired QoL in PCOS subjects, which is inconsistent with our results (60). However, it is worth noting that its inclusion criteria differ from ours. Besides, our findings should be cautiously explained due to the limited evidence.

The strengths of our analysis are that it was the first to look into the prevalence and severity of depression and anxiety targeting adolescent girls with PCOS, considering that adolescence is both an early-onset stage of these disorders and a critical period of personal development. We conducted a comprehensive literature search and included studies from multiple regions worldwide, which enhanced our results’ generalizability. Several limitations of our study should be considered. Firstly, the number of studies included was relatively small, and we didn’t delve deeper into the relationship between depression, anxiety, and potential factors. Second, most subjects were Caucasian, implying that more relevant studies should be conducted in more countries. Thirdly, according to the funnel plots (**Supplementary Figure S3**), it was suggested that there may be some publication bias. In addition, our study still had considerable heterogeneity despite employing appropriate statistical models. Finally, most of the studies we included were cross-sectional, which made it hard to clarify causality.

In conclusion, based on the available evidence, there was a significant association between PCOS and depression among adolescent girls in our study. More large-scale, well-designed studies are needed in the future to draw a more robust conclusion and to clarify the mechanisms linking PCOS and psychological distress. Clinicians should also pay more attention to the mental health status of PCOS patients to facilitate early recognition and timely intervention.

## Data Availability

The original contributions presented in the study are included in the article/**Supplementary Material**. Further inquiries can be directed to the corresponding authors.
