# Supplementary material for: Depression, anxiety and self-esteem in adolescent girls with polycystic ovary syndrome: a systematic review and meta-analysis
Source: Front Endocrinol (Lausanne). 2024 Sep 30;15:1399580. doi: 10.3389/fendo.2024.1399580 (PMC11471625; doi:10.3389/fendo.2024.1399580)
Supplement: Supplementary file 1 [file DataSheet1.pdf]

Supplemental Table 1 PRISMA 2020 Checklist

Section and Topic	Item #	Checklist item	Location where item is reported
**TITLE**			
Title	1	Identify the report as a systematic review.	Page 1
**ABSTRACT**			
Abstract	2	See the PRISMA 2020 for Abstracts checklist.	Pages 2 and 3
**INTRODUCTION**			
Rationale	3	Describe the rationale for the review in the context of existing knowledge.	Pages 4 to 6
Objectives	4	Provide an explicit statement of the objective(s) or question(s) the review addresses.	Page 6
**METHODS**			
Eligibility criteria	5	Specify the inclusion and exclusion criteria for the review and how studies were grouped for the syntheses.	Pages 7 and 8
Information sources	6	Specify all databases, registers, websites, organisations, reference lists and other sources searched or consulted to identify studies. Specify the date when each source was last searched or consulted.	Page 7
Search strategy	7	Present the full search strategies for all databases, registers and websites, including any filters and limits used.	Page 7, 8, and Supplemental files
Selection process	8	Specify the methods used to decide whether a study met the inclusion criteria of the review, including how many reviewers screened each record and each report retrieved, whether they worked independently, and if applicable, details of automation tools used in the process.	Page 8
Data collection process	9	Specify the methods used to collect data from reports, including how many reviewers collected data from each report, whether they worked independently, any processes for obtaining or confirming data from study investigators, and if applicable, details of automation tools used in the process.	Page 8
Data items	10a	List and define all outcomes for which data were sought. Specify whether all results that were compatible with each outcome domain in each study were sought (e.g. for all measures, time points, analyses), and if not, the methods used to decide which results to collect.	Pages 8 and 9
	10b	List and define all other variables for which data were sought (e.g. participant and intervention characteristics, funding sources). Describe any assumptions made about any missing or unclear information.	Page 8
Study risk of bias assessment	11	Specify the methods used to assess risk of bias in the included studies, including details of the tool(s) used, how many reviewers assessed each study and whether they worked independently, and if applicable, details of automation tools used in the process.	Page 9
Effect measures	12	Specify for each outcome the effect measure(s) (e.g. risk ratio, mean difference) used in the synthesis or presentation of results.	Page 9
Synthesis methods	13a	Describe the processes used to decide which studies were eligible for each synthesis (e.g. tabulating the study intervention characteristics and comparing against the planned groups for each synthesis (item #5)).	None
	13b	Describe any methods required to prepare the data for presentation or synthesis, such as handling of missing summary statistics, or data conversions.	None
	13c	Describe any methods used to tabulate or visually display results of individual studies and syntheses.	Page 9
	13d	Describe any methods used to synthesize results and provide a rationale for the choice(s). If meta-analysis was performed, describe the model(s), method(s) to identify the presence and extent of statistical heterogeneity, and software package(s) used.	Pages 9
	13e	Describe any methods used to explore possible causes of heterogeneity among study results (e.g. subgroup analysis, meta-regression).	Pages 9
	13f	Describe any sensitivity analyses conducted to assess robustness of the synthesized results.	None

Reporting bias assessment	14	Describe any methods used to assess risk of bias due to missing results in a synthesis (arising from reporting biases).	Pages 9
Certainty assessment	15	Describe any methods used to assess certainty (or confidence) in the body of evidence for an outcome.	Pages 9
**RESULTS**			
Study selection	16a	Describe the results of the search and selection process, from the number of records identified in the search to the number of studies included in the review, ideally using a flow diagram.	Page 10<br>Figure 1
	16b	Cite studies that might appear to meet the inclusion criteria, but which were excluded, and explain why they were excluded.	Figure 1
Study characteristics	17	Cite each included study and present its characteristics.	Pages 10 and 11<br>Table 1
Risk of bias in studies	18	Present assessments of risk of bias for each included study.	Table 1
Results of individual studies	19	For all outcomes, present, for each study: (a) summary statistics for each group (where appropriate) and (b) an effect estimate and its precision (e.g. confidence/credible interval), ideally using structured tables or plots.	Figures 2 to 5
Results of syntheses	20a	For each synthesis, briefly summarise the characteristics and risk of bias among contributing studies.	Pages 11 to 13
	20b	Present results of all statistical syntheses conducted. If meta-analysis was done, present for each the summary estimate and its precision (e.g. confidence/credible interval) and measures of statistical heterogeneity. If comparing groups, describe the direction of the effect.	Pages 11 to 13, Figures 2 to 5
	20c	Present results of all investigations of possible causes of heterogeneity among study results.	Page 13, Supplemental Fig S1 and S2
	20d	Present results of all sensitivity analyses conducted to assess the robustness of the synthesized results.	None
Reporting biases	21	Present assessments of risk of bias due to missing results (arising from reporting biases) for each synthesis assessed.	Pages 17 and 18, Supplemental Fig S3
Certainty of evidence	22	Present assessments of certainty (or confidence) in the body of evidence for each outcome assessed.	Pages 11 to 13
**DISCUSSION**			
Discussion	23a	Provide a general interpretation of the results in the context of other evidence.	Pages 13 to 17
	23b	Discuss any limitations of the evidence included in the review.	Pages 17 and 18
	23c	Discuss any limitations of the review processes used.	Pages 17 and 18
	23d	Discuss implications of the results for practice, policy, and future research.	Page 18
**OTHER INFORMATION**			
Registration and	24a	Provide registration information for the review, including register name and registration number, or state that the review was not registered.	Page 7

protocol	24b	Indicate where the review protocol can be accessed, or state that a protocol was not prepared.	Page 7
	24c	Describe and explain any amendments to information provided at registration or in the protocol.	None
Support	25	Describe sources of financial or non-financial support for the review, and the role of the funders or sponsors in the review.	Pages 18 and 19
Competing interests	26	Declare any competing interests of review authors.	Page 19
Availability of data, code and other materials	27	Report which of the following are publicly available and where they can be found: template data collection forms; data extracted from included studies; data used for all analyses; analytic code; any other materials used in the review.	Page 19

Supplemental Table 2 PRISMA 2020 for Abstracts Checklist

Section and Topic	Item #	Checklist item	Reported (Yes/No)
**TITLE**			
Title	1	Identify the report as a systematic review.	Yes
**BACKGROUND**			
Objectives	2	Provide an explicit statement of the main objective(s) or question(s) the review addresses.	Yes
**METHODS**			
Eligibility criteria	3	Specify the inclusion and exclusion criteria for the review.	Yes
Information sources	4	Specify the information sources (e.g. databases, registers) used to identify studies and the date when each was last searched.	Yes
Risk of bias	5	Specify the methods used to assess risk of bias in the included studies.	Yes
Synthesis of results	6	Specify the methods used to present and synthesise results.	Yes
**RESULTS**			
Included studies	7	Give the total number of included studies and participants and summarise relevant characteristics of studies.	Yes
Synthesis of results	8	Present results for main outcomes, preferably indicating the number of included studies and participants for each. If meta-analysis was done, report the summary estimate and confidence/credible interval. If comparing groups, indicate the direction of the effect (i.e. which group is favoured).	Yes
**DISCUSSION**			
Limitations of evidence	9	Provide a brief summary of the limitations of the evidence included in the review (e.g. study risk of bias, inconsistency and imprecision).	Yes
Interpretation	10	Provide a general interpretation of the results and important implications.	Yes
**OTHER**			
Funding	11	Specify the primary source of funding for the review.	No
Registration	12	Provide the register name and registration number.	Yes

From: Page MJ, McKenzie JE, Bossuyt PM, Boutron I, Hoffmann TC, Mulrow CD, et al. The PRISMA 2020 statement: an updated guideline for reporting systematic reviews. BMJ 2021;372:n71. doi: 10.1136/bmj.n71

Search strategies

232 articles were retrieved by PubMed (search date 2023/12/31)
244 articles were retrieved by PubMed (Update 2024/08/25)

#1 ((((((((((((((("Polycystic Ovary Syndrome"[MeSH Terms]) OR ("Ovary Syndrome, Polycystic"[Title/Abstract])) OR ("Syndrome, Polycystic Ovary"[Title/Abstract])) OR ("Stein-Leventhal Syndrome"[Title/Abstract])) OR ("Stein Leventhal Syndrome"[Title/Abstract])) OR ("Syndrome, Stein-Leventhal"[Title/Abstract])) OR ("Sclerocystic Ovarian Degeneration"[Title/Abstract])) OR ("Sclerocystic Ovary Syndrome"[Title/Abstract])) OR ("Polycystic Ovarian Syndrome"[Title/Abstract])) OR ("Ovarian Syndrome, Polycystic"[Title/Abstract])) OR ("Polycystic Ovary Syndrome 1"[Title/Abstract])) OR ("Sclerocystic Ovaries"[Title/Abstract])) OR ("Sclerocystic Ovary"[Title/Abstract])) OR ("PCOS"[Title/Abstract])) OR ("PCO"[Title/Abstract])) OR ("PCOD"[Title/Abstract])) OR ("ovary polycystic disease"[Title/Abstract]) 29431-30788

#2 ((((((((((((("Adolescent"[MeSH Terms]) OR ("Adolescents"[Title/Abstract])) OR ("Adolescence"[Title/Abstract])) OR ("Teens"[Title/Abstract])) OR ("Teen"[Title/Abstract])) OR ("Teenagers"[Title/Abstract])) OR ("Teenager"[Title/Abstract])) OR ("Youth"[Title/Abstract])) OR ("Youths"[Title/Abstract])) OR ("Adolescents, Female"[Title/Abstract])) OR ("Adolescent, Female"[Title/Abstract])) OR ("Female Adolescent"[Title/Abstract])) OR ("Female Adolescents"[Title/Abstract])) OR ("young"[Title/Abstract]) 2761866-2819449

#3 (((((("Depression"[MeSH Terms]) OR ("Depressive Symptoms"[Title/Abstract])) OR ("Depressive Symptom"[Title/Abstract])) OR ("Symptom, Depressive"[Title/Abstract])) OR ("Symptoms, Depressive"[Title/Abstract])) OR ("Emotional Depression"[Title/Abstract])) OR ("Depression, Emotional"[Title/Abstract]) 191044-199379

#4 (((((((("Anxiety"[MeSH Terms]) OR ("Angst"[Title/Abstract])) OR ("Social Anxiety"[Title/Abstract])) OR ("Anxieties, Social"[Title/Abstract])) OR ("Anxiety, Social"[Title/Abstract])) OR ("Social Anxieties"[Title/Abstract])) OR ("Hypervigilance"[Title/Abstract])) OR ("Nervousness"[Title/Abstract])) OR ("Anxiousness"[Title/Abstract]) 124021-129601

#5 ((((((((((((((((("Mental Disorders"[MeSH Terms]) OR ("Mental Disorder"[Title/Abstract])) OR ("Psychiatric Illness"[Title/Abstract])) OR ("Psychiatric Illnesses"[Title/Abstract])) OR ("Psychiatric Diseases"[Title/Abstract])) OR ("Psychiatric Disease"[Title/Abstract])) OR ("Mental Illness"[Title/Abstract])) OR ("Illness, Mental"[Title/Abstract])) OR ("Mental Illnesses"[Title/Abstract])) OR ("Psychiatric Disorders"[Title/Abstract])) OR ("Psychiatric Disorder"[Title/Abstract])) OR ("Behavior Disorders"[Title/Abstract])) OR ("Diagnosis, Psychiatric"[Title/Abstract])) OR ("Psychiatric Diagnosis"[Title/Abstract])) OR ("Mental Disorders, Severe"[Title/Abstract])) OR ("Mental Disorder, Severe"[Title/Abstract])) OR ("Severe Mental Disorder"[Title/Abstract])) OR ("Severe Mental Disorders"[Title/Abstract])) OR ("mental disease"[Title/Abstract]) 1512645-1550862

#6 ((((((("Mood Disorders"[MeSH Terms]) OR ("Disorder, Mood"[Title/Abstrac

t])) OR ("Disorders, Mood"[Title/Abstract])) OR ("Mood Disorder"[Title/Abstract])) OR ("Affective Disorders"[Title/Abstract])) OR ("Affective Disorder"[Title/Abstract])) OR ("Disorder, Affective"[Title/Abstract])) OR ("Disorders, Affective"[Title/Abstract]) 183506-186985

#7 (((((("Psychological Distress"[MeSH Terms]) OR ("distress syndrome"[Title/Abstract])) OR ("Distress, Psychological"[Title/Abstract])) OR ("Emotional Distress"[Title/Abstract])) OR ("Distress, Emotional"[Title/Abstract])) OR ("Emotional Stress"[Title/Abstract])) OR ("Stress, Emotional"[Title/Abstract]) 59818-62494

#8 (((("Mental Health"[MeSH Terms])) OR ("Health, Mental"[Title/Abstract])) OR ("Mental Hygiene"[Title/Abstract])) OR ("Hygiene, Mental"[Title/Abstract]) 66802-70909

#9 ((((("Emotions"[MeSH Terms]) OR ("Emotion"[Title/Abstract])) OR ("Regret"[Title/Abstract])) OR ("Regrets"[Title/Abstract])) OR ("Feelings"[Title/Abstract])) OR ("Feeling"[Title/Abstract]) 512429-532810

#10 #3 OR #4 OR #5 OR #6 OR #7 OR #8 OR #9 1974470-2033281

#11 #1 AND #2 AND #10 **232-244**

477 articles were retrieved by Web of Science (search date 2023/12/31)
515 articles were retrieved by Web of Science (Update 2024/08/25)

#1 TS=("Polycystic Ovary Syndrome") OR AB=("Ovary Syndrome, Polycystic" OR "Syndrome, Polycystic Ovary" OR "Stein-Leventhal Syndrome" OR "Stein Leventhal Syndrome" OR "Syndrome, Stein-Leventhal" OR "Sclerocystic Ovarian Degeneration" OR "Ovarian Degeneration, Sclerocystic" OR "Sclerocystic Ovary Syndrome" OR "Polycystic Ovarian Syndrome" OR "Ovarian Syndrome, Polycystic" OR "Polycystic Ovary Syndrome 1" OR "Sclerocystic Ovaries" OR "Ovary, Sclerocystic" OR "Sclerocystic Ovary" OR "PCOS" OR "PCO" OR "PCOD" OR "ovary polycystic disease") 51833-54196

#2 TS=("Adolescent") OR AB=("Adolescents" OR "Adolescence" OR "Teens" OR "Teen" OR "Teenagers" OR "Teenager" OR "Youth" OR "Youths" OR "Adolescents, Female" OR "Adolescent, Female" OR "Female Adolescent" OR "Female Adolescents" OR "young") 3774931-3866191

#3 TS=("Depression") OR AB=("Depressive Symptoms" OR "Depressive Symptom" OR "Symptom, Depressive" OR "Symptoms, Depressive" OR "Emotional Depression" OR "Depression, Emotional") 953533-989856

#4 TS=("Anxiety") OR AB=("Angst" OR "Social Anxiety" OR "Anxieties, Social" OR "Anxiety, Social" OR "Social Anxieties" OR "Hypervigilance" OR "Nervousness" OR "Anxiousness") 590618-618617

#5 TS=("Mental Disorders") OR AB=("Mental Disorder" OR "Psychiatric Illness" OR "Psychiatric Illnesses" OR "Psychiatric Diseases" OR "Psychiatric Disease" OR "Mental Illness" OR "Illness, Mental" OR "Mental Illnesses" OR "Psychiatric Disorders" OR "Psychiatric Disorder" OR "Behavior Disorders" OR "Diagnosis, Psychiatric" OR "Psychiatric Diagnosis" OR "Mental Disorders, Severe" OR "Mental Disorder, Severe" OR "Severe Mental Disorder" OR "Severe Ment

al Disorders" OR "mental disease") 1330625-1382038
#6 TS=("Mood Disorders") OR AB=("Disorder, Mood" OR "Disorders, Mood" OR "Mood Disorder" OR "Affective Disorders" OR "Affective Disorder" OR " Disorder, Affective" OR "Disorders, Affective") 68190-70226
#7 TS=("Psychological Distress") OR AB=("distress syndrome" OR "Distress, Psychological" OR "Emotional Distress" OR "Distress, Emotional" OR "Emotional Stress" OR "Stress, Emotional") 113464-118972
#8 TS=("Mental Health") OR AB=("Health, Mental" OR "Mental Hygiene" OR "Hygiene, Mental") 507515-543245
#9 TS=("Emotions") OR AB=("Emotion" OR "Regret" OR "Regrets" OR "Feelings" OR "Feeling") 529253-550784
#10 #3 OR #4 OR #5 OR #6 OR #7 OR #8 OR #9 2988677-3104340
#11 #1 AND #2 AND #10 **477-515**

850 articles were retrieved by Embase (search date 2023/12/31)
924 articles were retrieved by Embase (Update 2024/08/25)
#1 'ovary polycystic disease'/exp OR 'ovary syndrome, polycystic':ab,ti OR 'syndrome, polycystic ovary':ab,ti OR 'stein-leventhal syndrome':ab,ti OR 'stein leventhal syndrome':ab,ti OR 'syndrome, stein-leventhal':ab,ti OR 'sclerocystic ovarian degeneration':ab,ti OR 'ovarian degeneration, sclerocystic':ab,ti OR 'sclerocystic ovary syndrome':ab,ti OR 'polycystic ovarian syndrome':ab,ti OR 'ovarian syndrome, polycystic':ab,ti OR 'polycystic ovary syndrome 1':ab,ti OR 'sclerocystic ovaries':ab,ti OR 'ovary, sclerocystic':ab,ti OR 'sclerocystic ovary':ab,ti OR 'pcos':ab,ti OR 'pco':ab,ti OR 'pcod':ab,ti OR 'polycystic ovary syndrome':ab,ti 59341-61826
#2 'adolescent'/exp OR 'adolescents':ab,ti OR 'adolescence':ab,ti OR 'teens':ab,ti OR 'teen':ab,ti OR 'teenagers':ab,ti OR 'teenager':ab,ti OR 'youth':ab,ti OR 'youths':ab,ti OR 'adolescents, female':ab,ti OR 'adolescent, female':ab,ti OR 'female adolescent':ab,ti OR 'female adolescents':ab,ti OR 'young':ab,ti 2687122-2769272
#3 'depression'/exp OR 'depressive symptoms':ab,ti OR 'depressive symptom':ab,ti OR 'symptom, depressive':ab,ti OR 'symptoms, depressive':ab,ti OR 'emotional depression':ab,ti OR 'depression, emotional':ab,ti 669708-705198
#4 'anxiety'/exp OR 'angst':ab,ti OR 'social anxiety':ab,ti OR 'anxieties, social':ab,ti OR 'anxiety, social':ab,ti OR 'social anxieties':ab,ti OR 'hypervigilance':ab,ti OR 'nervousness':ab,ti OR 'anxiousness':ab,ti 317126-338091
#5 'mental disease'/exp OR 'mental disorder':ab,ti OR 'psychiatric illness':ab,ti OR 'psychiatric illnesses':ab,ti OR 'psychiatric diseases':ab,ti OR 'psychiatric disease':ab,ti OR 'mental illness':ab,ti OR 'illness, mental':ab,ti OR 'mental illnesses':ab,ti OR 'psychiatric disorders':ab,ti OR 'psychiatric disorder':ab,ti OR 'behavior disorders':ab,ti OR 'diagnosis, psychiatric':ab,ti OR 'psychiatric diagnosis':ab,ti OR 'mental disorders, severe':ab,ti OR 'mental disorder, severe':ab,ti OR 'severe mental disorder':ab,ti OR 'severe mental disorders':ab,ti OR 'mental disorders':ab,

ti 2946044-3059800
#6 'mood disorder'/exp OR 'disorder, mood':ab,ti OR 'disorders, mood':ab,ti OR 'mood disorders':ab,ti OR 'affective disorders':ab,ti OR 'affective disorder':ab,ti OR 'disorder, affective':ab,ti OR 'disorders, affective':ab,ti 718738-755355
#7 'distress syndrome'/exp OR 'psychological distress':ab,ti OR 'distress, psychological':ab,ti OR 'emotional distress':ab,ti OR 'distress, emotional':ab,ti OR 'emotional stress':ab,ti OR 'stress, emotional':ab,ti 100345-105975
#8 'mental health'/exp OR 'health, mental':ab,ti OR 'mental hygiene':ab,ti OR 'hygiene, mental':ab,ti 251006-272250
#9 'emotion'/exp OR 'emotions':ab,ti OR 'regret':ab,ti OR 'regrets':ab,ti OR 'feelings':ab,ti OR 'feeling':ab,ti 925840-977198
#10 #3 OR #4 OR #5 OR #6 OR #7 OR #8 OR #9 3570867-3717040
#11 #1 AND #2 AND #10 **850-924**

2 articles were retrieved by Cochrane Reviews database (search date 2023/12/31)
2 articles were retrieved by Cochrane Reviews database (Update 2024/08/25)
#**1** MeSH descriptor: [Polycystic Ovary Syndrome] explode all trees 1935-2164
#**2** ("Ovary Syndrome, Polycystic" OR "Syndrome, Polycystic Ovary" OR "Stein-Leventhal Syndrome" OR "Stein Leventhal Syndrome" OR "Syndrome, Stein-Leventhal" OR "Sclerocystic Ovarian Degeneration" OR "Ovarian Degeneration, Sclerocystic" OR "Sclerocystic Ovary Syndrome" OR "Polycystic Ovarian Syndrome" OR "Ovarian Syndrome, Polycystic" OR "Polycystic Ovary Syndrome 1" OR "Sclerocystic Ovaries" OR "Ovary, Sclerocystic" OR "Sclerocystic Ovary" OR "PCOS" OR "PCO" OR "PCOD" OR "ovary polycystic disease"):ti,ab,kw (Word variations have been searched) 5343-5599
#**3** #1 OR #2 5534-5792
#**4** MeSH descriptor: [Adolescent] explode all trees 126257-137774
#**5** ("Adolescents" OR "Adolescence" OR "Teens" OR "Teen" OR "Teenagers" OR "Teenager" OR "Youth" OR "Youths" OR "Adolescents, Female" OR "Adolescent, Female" OR "Female Adolescent" OR "Female Adolescents" OR "young"):ti,ab,kw (Word variations have been searched) 249713-269431
#**6** #4 OR #5 249713-269431
#**7** MeSH descriptor: [Depression] explode all trees 18859-18451
#**8** ("Depressive Symptoms" OR "Depressive Symptom" OR "Symptom, Depressive" OR "Symptoms, Depressive" OR "Emotional Depression" OR "Depression, Emotional"):ti,ab,kw (Word variations have been searched) 18369-19817
#**9** #7 OR #8 31512-32559
#**10** MeSH descriptor: [Anxiety] explode all trees 13405-12844
#**11** ("Angst" OR "Social Anxiety" OR "Anxieties, Social" OR "Anxiety, Social" OR "Social Anxieties" OR "Hypervigilance" OR "Nervousness" OR "Anxiousness"):ti,ab,kw (Word variations have been searched) 37561-39976
#**12** #10 OR #11 49330-51240

#**13** MeSH descriptor: [Mental Disorders] explode all trees 100836-107768
#**14** ("Mental Disorder" OR "Psychiatric Illness" OR "Psychiatric Illnesses" OR "Psychiatric Diseases" OR "Psychiatric Disease" OR "Mental Illness" OR "Illness, Mental" OR "Mental Illnesses" OR "Psychiatric Disorders" OR "Psychiatric Disorder" OR "Behavior Disorders" OR "Diagnosis, Psychiatric" OR "Psychiatric Diagnosis" OR "Mental Disorders, Severe" OR "Mental Disorder, Severe" OR "Severe Mental Disorder" OR "Severe Mental Disorders" OR "mental disease"):ti,ab,kw (Word variations have been searched) 31422-33362
#**15** #13 OR #14 119476-127426
#**16** MeSH descriptor: [Mood Disorders] explode all trees 19079-20393
#**17** ("Disorder, Mood" OR "Disorders, Mood" OR "Mood Disorder" OR "Affective Disorders" OR "Affective Disorder" OR "Disorder, Affective" OR "Disorders, Affective"):ti,ab,kw (Word variations have been searched) 6503-6882
#**18** #16 OR #17 23452-25014
#**19** MeSH descriptor: [Psychological Distress] explode all trees 435-529
#**20** ("distress syndrome" OR "Distress, Psychological" OR "Emotional Distress" OR "Distress, Emotional" OR "Emotional Stress" OR "Stress, Emotional"):ti,ab,kw (Word variations have been searched) 13932-14899
#**21** #19 OR #20 14201-15194
#**22** MeSH descriptor: [Mental Health] explode all trees 3670-3319
#**23** ("Health, Mental" OR "Mental Hygiene" OR "Hygiene, Mental"):ti,ab,kw (Word variations have been searched) 1094-1278
#**24** #22 OR #23 4559-4404
#**25** MeSH descriptor: [Emotions] explode all trees 40227-40247
#**26** ("Emotion" OR "Regret" OR "Regrets" OR "Feelings" OR "Feeling"):ti,ab,kw (Word variations have been searched) 54027-58759
#**27** #25 OR #26 84615-88862
#**28** #9 OR #12 OR #15 OR #18 OR #21 OR #24 OR #27 227007-240487
#**29** #3 AND #6 AND #28 in Cochrane Reviews **2-2**

Subgroup analyses

Fig S1 Subgroup analyses by continent of origin

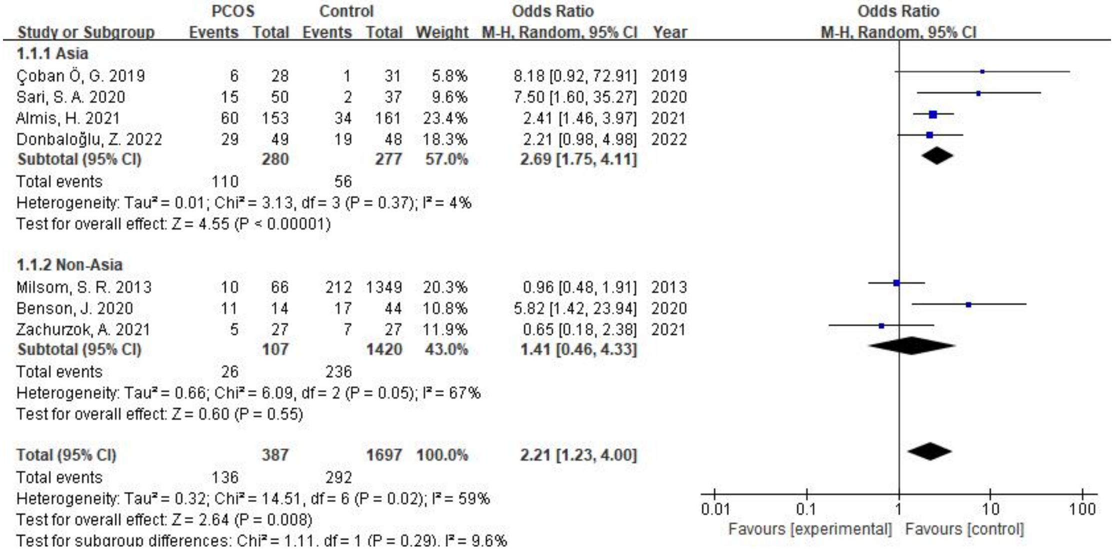

(a). the prevalence of depression

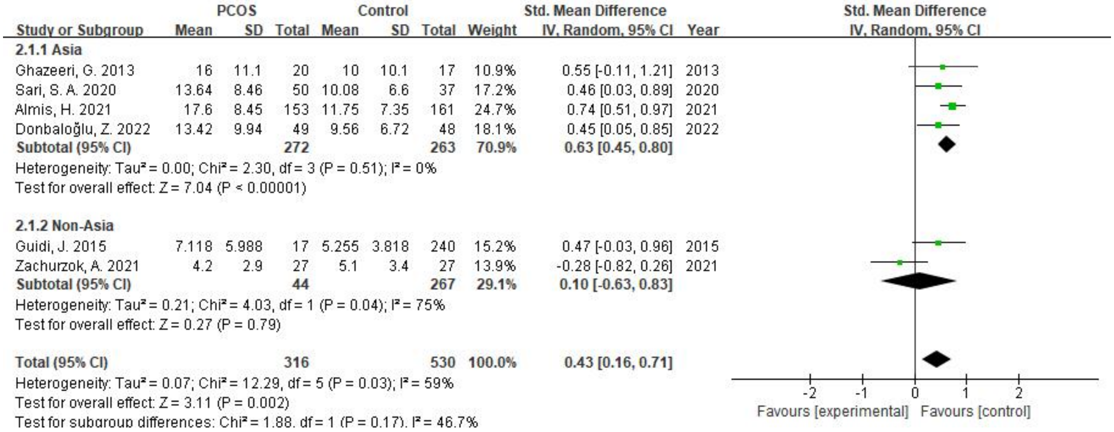

(b). depression scores

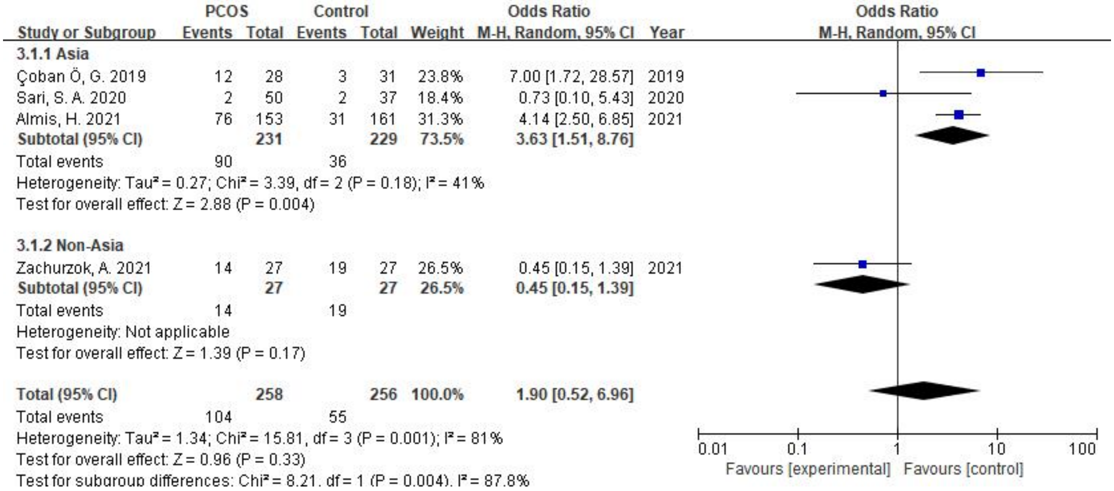

(c). the prevalence of anxiety

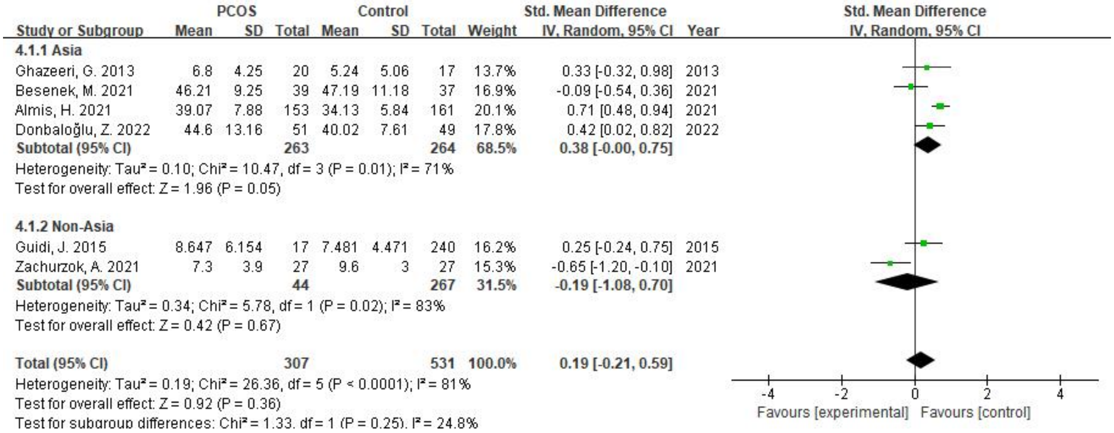

(d). anxiety scores

Fig S2 Subgroup analyses by screening tool

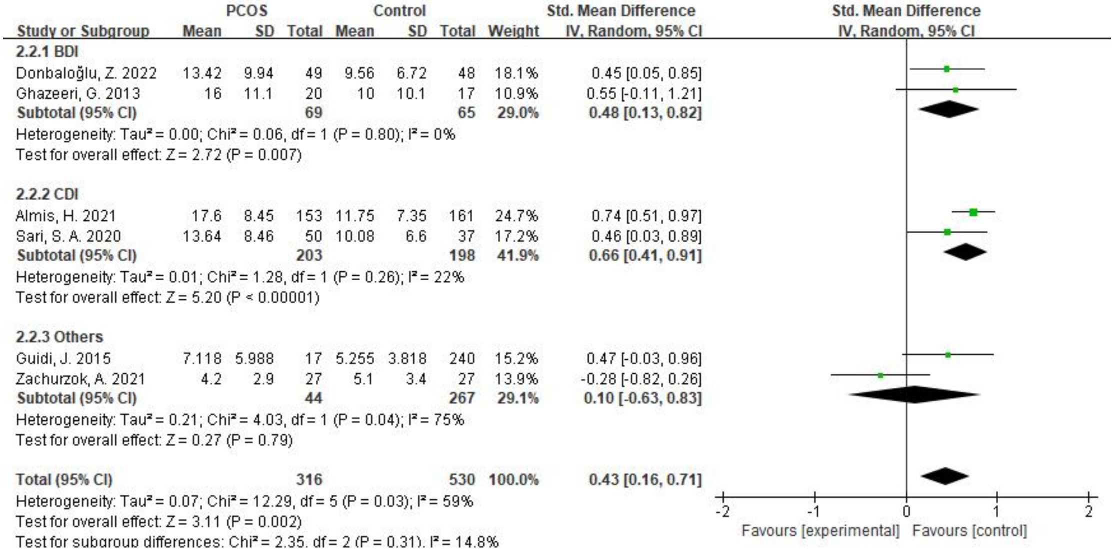

(a). depression scores

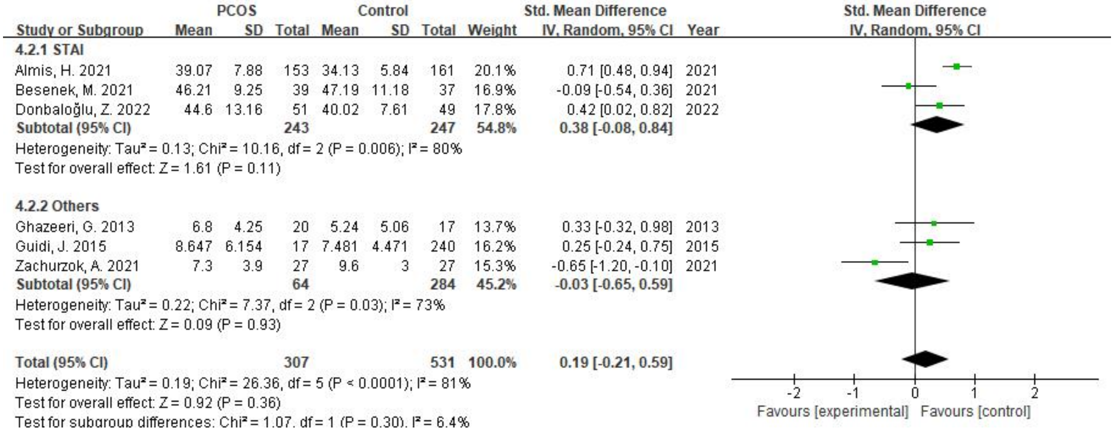

(b). anxiety scores

Fig S3 Funnel plots

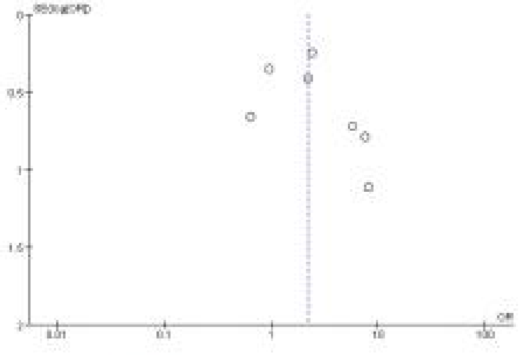

(a). the prevalence of depression

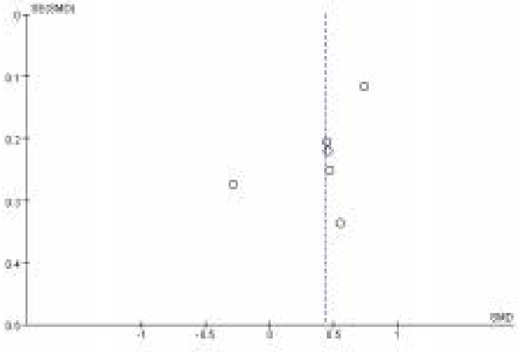

(b). depression scores

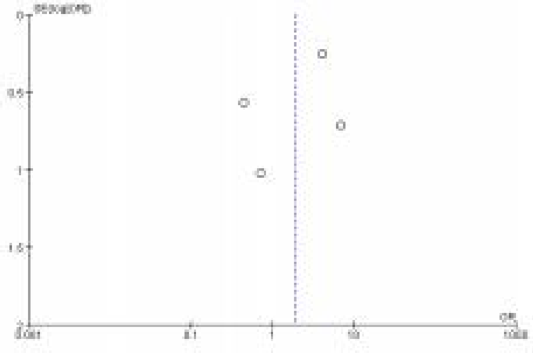

(c). the prevalence of anxiety

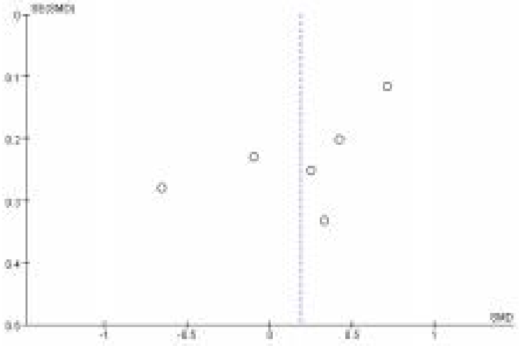

(d). anxiety scores

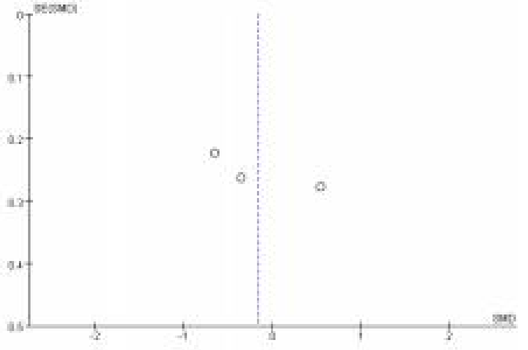

(e). self-esteem scores

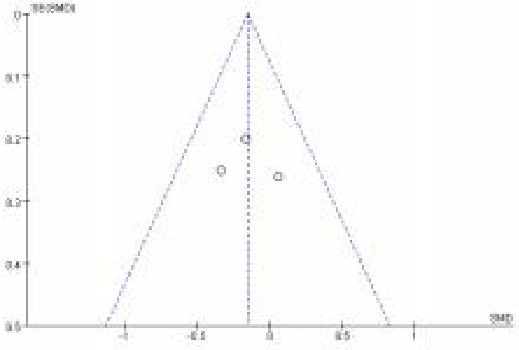

(f). quality of life scores
